# HIF-1 inhibition reverses opacity in a rat model of galactose-induced cataract

**DOI:** 10.1371/journal.pone.0299145

**Published:** 2024-02-28

**Authors:** Masaru Takashima, Masaya Nagaya, Yoshihiro Takamura, Masaru Inatani, Masaya Oki

**Affiliations:** 1 Department of Industrial Creation Engineering, Graduate School of Engineering, University of Fukui, Fukui, Japan; 2 Department of Ophthalmology, Faculty of Medical Sciences, University of Fukui, Fukui, Japan; 3 Life Science Innovation Center, University of Fukui, Fukui, Japan; University of Colorado Denver School of Medicine, UNITED STATES

## Abstract

Cataract is an eye disease, in which the lens becomes opaque, causing vision loss and blindness. The detailed mechanism of cataract development has not been characterized, and effective drug therapies remain unavailable. Here, we investigated the effects of Hypoxia-inducible factor 1 (HIF-1) inhibitors using an *ex vivo* model, in which rat lenses were cultured in galactose-containing medium to induce opacity formation. We found that treatment with the HIF-1 inhibitors 2-Methoxyestradiol (2ME2), YC-1, and Bavachinin decreased lens opacity. Microarray analysis on 2ME2-treated samples, in which opacity was decreased, identified genes upregulated by galactose and downregulated by inhibitor treatment. Subsequent STRING analysis on genes that showed expression change by RT-qPCR identified two clusters. First cluster related to the cytoskeleton and epithelial-mesenchymal transition (EMT). Second cluster related to the oxidative stress, and apoptosis. ACTA2, a known marker for EMT, and TXNIP, a suppressor of cell proliferation and activator of apoptosis, were present in each cluster. Thus, suppression of EMT and apoptosis, as well as activation of cell proliferation, appear to underlie the decrease in lens opacity.

## Introduction

Cataract is an eye disease, in which the lens becomes opaque, resulting in vision loss and blindness [1]. Currently, the only treatment for cataract is surgical lens replacement, which is not always broadly accessible in developing countries [2]. Therefore, the development of new drugs for cataract treatment is desired.

Factors that promote cataract formation include aging, ultraviolet light, and diabetes. Among these, diabetes is of particular interest because the number of diabetic patients is increasing yearly, and thus the risk of developing cataracts even at a younger age is high [3]. Therefore, we focused our study on diabetic cataracts.

Both *in vivo* and *ex vivo* animal models of diabetic cataract have been established. In the STZ *in vivo* model, administration of streptozotocin induces type-I diabetes [4] and leads to cataracts, whereas the Nile grass rat is a model for cataract development as a result of type-II diabetes [5]. In the galactose diet-loaded model, cataracts are induced by placing rats on a galactose-containing diet [6]. Cataracts are induced in a similar manner in an *ex vivo* galactose-induced cataract model, in which rat lenses are cultured in galactose-containing medium to induce opacity [7]. As this *ex vivo* model can rapidly and stably induce opacity, making it useful for drug screening, it was also used in this study.

Age-related cataracts tend to develop opacity in the lens nucleus, whereas diabetic cataracts typically develop opacity in the lens cortex [8]. The mechanisms of diabetic cataract formation include osmotic stress, non-enzymatic glycation, and oxidative stress [9].

Osmotic stress is caused by the accumulation of sugar alcohols through the action of aldose reductase (AR) [10]. Under hyperglycemic conditions, AR reduces sugars to sugar alcohols, which are less membrane-permeable, not easily metabolized, and accumulate in lens fibers, thus increasing intracellular osmotic pressure. This hyperosmotic environment absorbs water, causes lens fibers to swell, and results in opacity.

In addition, methylglyoxal, produced by the non-enzymatic glycation of sugar metabolic intermediates in a high-sugar environment binds various proteins and impairs their function. Therefore, abnormal protein aggregation also results in opacity [11].

Oxidative stress leads to opacity when accumulated reactive oxygen species (ROS), such as hydrogen peroxide, cause protein denaturation and cellular damage [12]. Reduced glutathione (GSH) acts as a reducing agent to remove ROS, and oxidized glutathione (GSSG) is reduced to GSH using NADPH as a coenzyme. However, since AR also uses NADPH to convert glucose to sorbitol, cellular NADPH becomes depleted, inhibiting GSSG reduction and promoting oxidative stress [13].

Furthermore, recent reports suggest the involvement of lens epithelial cell (LEC) apoptosis and epithelial-mesenchymal transition (EMT) in diabetic cataract formation [14, 15]. Thus, diabetic cataract may be caused by a disruption of LEC homeostasis.

Previously, we reported that polo like kinase 3 (PLK3) and ataxia telangiectasia mutated (ATM) are involved in galactose-induced cataract [16, 17]. The PLK3 inhibitor, GW843682X, prevented the formation of opacity in rat lenses cultured in galactose medium [16], and the ATM inhibitors, AZD0156 and KU55933, both prevented and reversed galactose-mediated opacity formation in rat lenses [17]. Further exploration of PLK3 and ATM downstream factors identified Hypoxia-inducible factor 1 (HIF-1), a protein induced under hypoxia [18–21].

HIF-1 is a heterodimer of HIF-1α and HIF-1β [22]. HIF-1 acts as a transcriptional activator of many genes involved in angiogenesis, glucose metabolism, cell proliferation, and invasion [23]. Under normoxia, the HIF-1α protein, a component of HIF-1, is hydroxylated by the oxygen substrate, prolyl hydroxylase-domain protein (PHD) [24, 25]. Hydroxylated HIF-1α is recognized by the von Hippel-Lindau (VHL) tumor-suppressor protein, which ubiquitinates HIF-1α, leading it to proteasomal degradation [26, 27]. Conversely, under hypoxia, HIF-1α, without PHD hydroxylation, translocates to the nucleus to form a heterodimer with HIF-1β. HIF-1 forms a complex with p300 and acts as a transcription factor [28]. The lens is maintained in a hypoxic state, and HIF-1α has been reported to promote organelle degradation within the lens [29]. Nevertheless, the relationship between HIF-1α and cataract remains unclear, with reports of HIF-1α to be promoting cataract and reducing its risk [30, 31]. Moreover, HIF-1α is stably expressed in a high-glucose environment, suggesting that it may also be induced in a galactose-rich environment [32].

In this study, we investigated whether galactose-induced opacity could be decreased by HIF-1 inhibitors. Using RT-qPCR, we examined the expression of genes inhibited by the addition of HIF-1 and identified genes involved in cataract formation. These results indicate that targeting HIF-1 and its downstream genes may be a useful approach to reversing lens opacity.

## Materials and methods

### Animals

Six-week-old male SD rats were purchased from Sankyo Laboratory Service (Japan) and used for the experiments. All experiments were approved by the Animal Research Committee of the University of Fukui (approval number: 28091) and conducted in accordance with the University of Fukui regulations on animal experiments and Association for Research in Vision and Ophthalmology Statement for the Use of Animals in Ophthalmic and Vision Research as described previously [17]. This study adhered to the ARRIVE guidelines in its reporting.

### *Ex vivo* assays

Rats were euthanized by CO_2_ asphyxiation and the lenses were removed. All lenses were incubated in 2 mL M199 medium containing 0.1% BSA and 30 mM galactose for 2–3 days in an incubator at 5% CO_2_ and 37°C to induce opacity, as described previously [16]. After opacity induction, images were acquired under a microscope, and one lens was incubated with 2 mL fresh medium supplemented by 16 μL DMSO, whereas the other was incubated with 2 mL fresh medium supplemented by 16 μL 2ME2 (Selleck Chemical, USA), a HIF-1 inhibitor, which was dissolved in DMSO at final concentrations of 10 μM, 20 μM, and 40 μM. The lens was incubated for an additional 2–3 days and imaged. Control samples, in which cataracts were not induced, were incubated for 4 or 6 days with sterile water instead of galactose.

### Microscopic observations

Lens images were obtained in a darkroom using an SZX12 stereomicroscope equipped with a DP58 camera (Olympus, Japan), as described previously [16]. Samples were imaged in 35-mm Petri dishes containing 7 mL PBS. The lens-cortex opacity was measured in ImageJ as the luminance (0–255) of the opaque lens area, and a weighted average was calculated [17].

### Microarray data analysis

Microarray analysis was performed from Control samples cultured for 4 or 6 days (n = 2), cataract-induced samples cultured in galactose medium for 6 days (n = 3), and samples treated with 2ME2 (n = 2). A GeneChip Rat Gene 2.0 ST array chip (Thermo Fisher Scientific, USA) was used for microarray experiments as described previously [16]. Preprocessing and data analyses were performed using the R software (Version 4.0.3). First, data from all samples were normalized using the Robust Multi-array Average algorithm, and probes not corresponding to genes were excluded. Next, genes with signal values of <5 were excluded from all sample datasets. Signal values for each condition were normalized to the mean value of replicate samples. Genes were selected as significant if their expression increased by more than two-fold between the Control and Galactose groups and decreased by more than two-fold between the galactose and 2ME2 groups. The extracted genes were subjected to functional analysis using STRING (https://version-11-5.string-db.org/). Microarray data are available in the GEO repository under the accession number GSE240617 (https://www.ncbi.nlm.nih.gov/geo/query/acc.cgi?acc=GSE240617).

### RNA extraction, cDNA preparation, and real-time RT-qPCR

Lens RNA extraction and real-time RT-qPCR were performed using the same methods as described previously [33]. The primers used are listed in S1 Table. Gene expression levels were normalized to *Gapdh* expression. To evaluate differences between the galactose group and the control (6Days cultured) and 2ME2 groups, genes with more than 10% decrease in expression from the galactose group to the 2ME2 group relative to the amount of increase from the control sample to the galactose sample were considered important genes.

## Results

### HIF-1 inhibitors decrease opacity of galactose-induced rat cataracts

**Fig 1:**
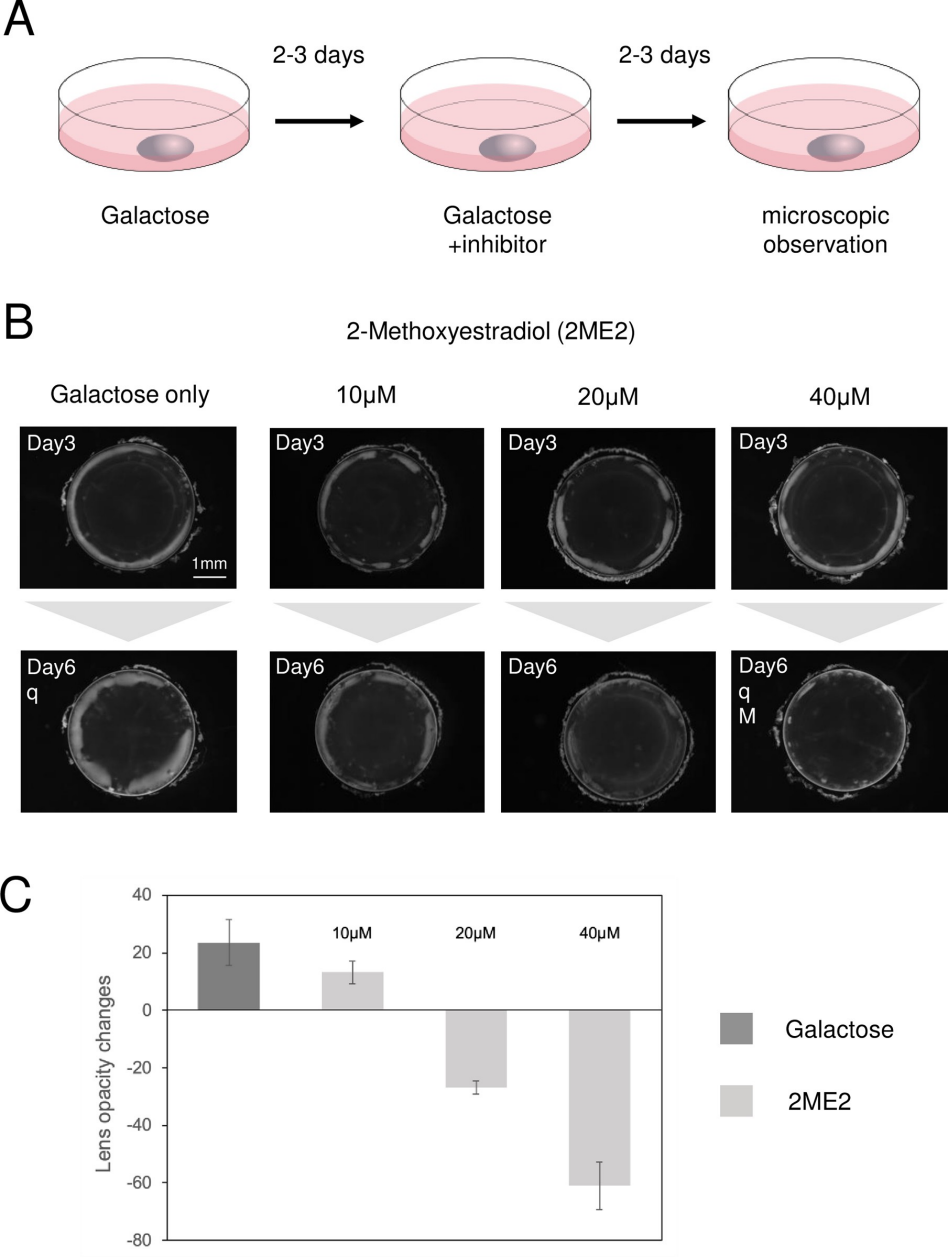
Effect of 2ME2 on lens opacity. (A) Schematic representation of the SD rat lens experiment. (B) Rat lenses were cultured in medium containing 30 mM galactose for 2–3 days (upper panel). After image acquisition, DMSO as a vehicle control or 10 μM, 20 μM, or 40 μM 2ME2 in DMSO were added to the galactose-containing medium, and culture was continued for 2–3 days. The number of days indicated in each panel represents the total days of incubation, “q” indicates samples used for RT-qPCR, and “M” indicates samples used for microarray analysis. (C) The level of lens opacity with and without 2ME2, as well as the change in opacity before and after addition of the inhibitor, were calculated [17]. Data are expressed as the mean ± SE. The two additional samples used for the quantification are shown in S2 Fig.

In a previous study, we reported that the ATM inhibitors, AZD0156 and KU55933, decreased opacity in a rat lens model of galactose-induced cataract [17]. ATM is reported to stabilize HIF-1α by phosphorylation [18]. Therefore, we investigated whether inhibiting HIF-1, which consists of HIF-1α and HIF-1β, would reverse the opacity phenotype. First, lenses were removed from Sprague-Dawley (SD) rats and cultured in galactose-containing medium for 2–3 days to form opacities in the equatorial cortex. Subsequently, the medium was changed, and lenses were cultured for an additional 2–3 days in medium containing only galactose or galactose supplemented by one of the ten HIF-1 inhibitors used (Fig 1A and 1B) [34–43]. The HIF-1 inhibitors and concentrations used are listed in Table 1. In the galactose-only medium, the opacity area was increased compared to that before medium exchange. 2-Methoxyestradiol (2ME2), YC-1, and Bavachinin were the only HIF-1 inhibitors that could decrease opacity (Fig 1B and S1 Fig). 2ME2 inhibits HIF-1α translation, YC-1 inhibits HIF-1 and p300 binding, and Bavachinin, is a PPAR-agonist that activates the interaction between VHL and HIF-1α [34–36]. In this study, we focused on 2ME2, which directly inhibits HIF-1α translation. We tested 10 μM, 20 μM, and 40 μM of 2ME2 and observed that only 20 μM and 40 μM resulted in a decrease in lens opacity (Fig 1B and S2 Fig). Next, we quantified the opacity change before and after 2ME2 administration (Fig 1C), as previously established [17]. The quantification is shown in Fig 1B and S2 Fig. As treatment with 40 μM 2ME2 resulted in the highest opacity decrease, this concentration was used in subsequent experiments.

**Table 1:** List of HIF-1 inhibitors used.

Name	Place of purchase	Concentration	Therapeutic effect	Source
2-Methoxyestradiol (2ME2)	Selleck Chemical (USA)	10, 20, 40μM	(20, 40μM)	(34)
YC-1	Wako (Japan)	25, 40, 50, 100μM	(25μM)	(35)
Bavachinin	Cayman Chemical (USA)	2.5, 5, 10, 20, 25, 50, 100μM	(2.5, 5, 10μM)	(36)
BAY87-2243	Selleck Chemical (USA)	10, 20, 40μM	×	(37)
Chetomin	Cayman Chemical (USA)	50nM	×	(38)
ELR510444	Cayman Chemical (USA)	0.1, 1, 10μM	×	(39)
KC7F2	Cayman Chemical (USA)	10, 20, 40, 80μM	×	(40)
PX-478	Cayman Chemical (USA)	20, 40, 80μM	×	(41)
Topotecan	Cayman Chemical (USA)	0.125, 0.5, 2μM	×	(42)
Vitexin	Cayman Chemical (USA)	25, 50μM	×	(43)

The list summarizes the Therapy effect of 10 HIF-1 inhibitors. Concentration indicates the concentration of the drug used. Therapeutic effect column, "○" indicates that the HIF-1 inhibitor had therapeutic effect, "×" indicates that the HIF-1 inhibitor had no therapeutic effect.

### Identification of genes involved in opacity decrease using microarray analysis

**Fig 2:**
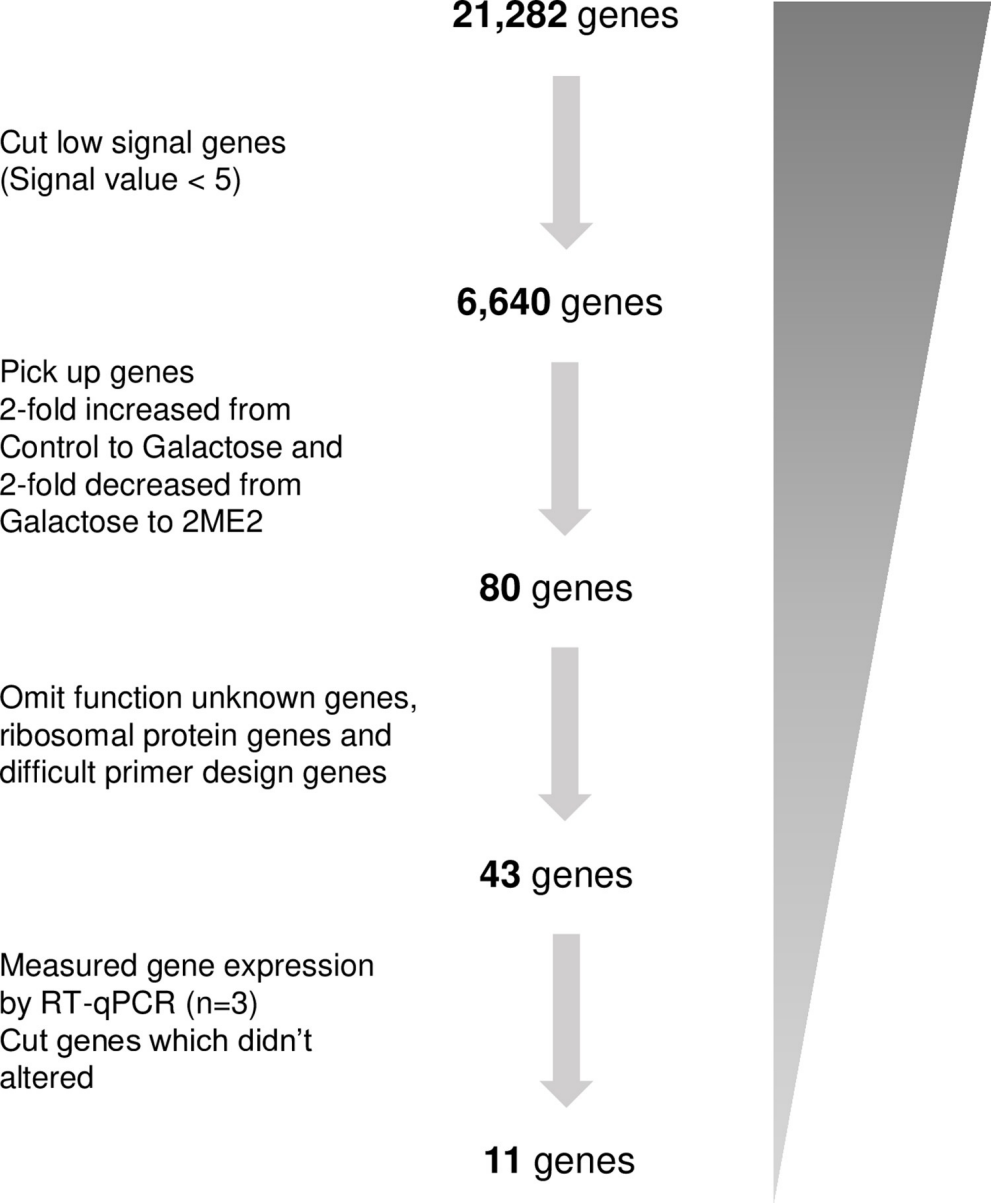
Flowchart for narrowing down the genes for which expression levels were altered upon 2ME2 addition. Probes without gene names were removed (21,282 genes remaining), genes with signal values of <5 were excluded (6,640 genes remaining), genes exhibiting more than two-fold increase between the Control and Galactose groups as well as decrease between the Galactose and 2ME2 groups were included (80 genes remaining), and genes of unknown function, ribosomal-protein genes, and genes for which primer design was difficult were excluded (43 genes remaining). Of these, 11 genes exhibited more than 10% expression decrease between the Galactose and 2ME2 groups compared with amount of increase expression between the Control and Galactose groups by RT-qPCR, which were consistent with the microarray analysis.

To identify genes involved in galactose-mediated opacity formation, as well as opacity reversal upon 2ME2 addition, we conducted microarray analysis on the following sample groups: Previous study used sample incubated for 4 days without galactose [16], this study added a sample incubated for 6 days without galactose, for a total of 2 samples (Control group), three samples cultured for 6 days in galactose-containing medium to induce opacification (Galactose group), and two samples cultured for 3 days in galactose-only medium and then a further 3 days in galactose medium supplemented by 2ME2 (2ME2 group). A flowchart of the extraction process is shown in Fig 2. First, after removing probes lacking gene names, out of the 36,685 probes, genes with signal values of <5 were excluded from the 21,282 genes to narrow down the number of genes to 6,640. Subsequently, the average signal values between the Control, Galactose, and 2ME2 groups were calculated. We identified 196 genes for which expression levels were increased by more than two-fold between the Control and Galactose groups and decreased by more than 1.5-fold between the Galactose and 2ME2 groups (S1 Dataset).

**Fig 3:**
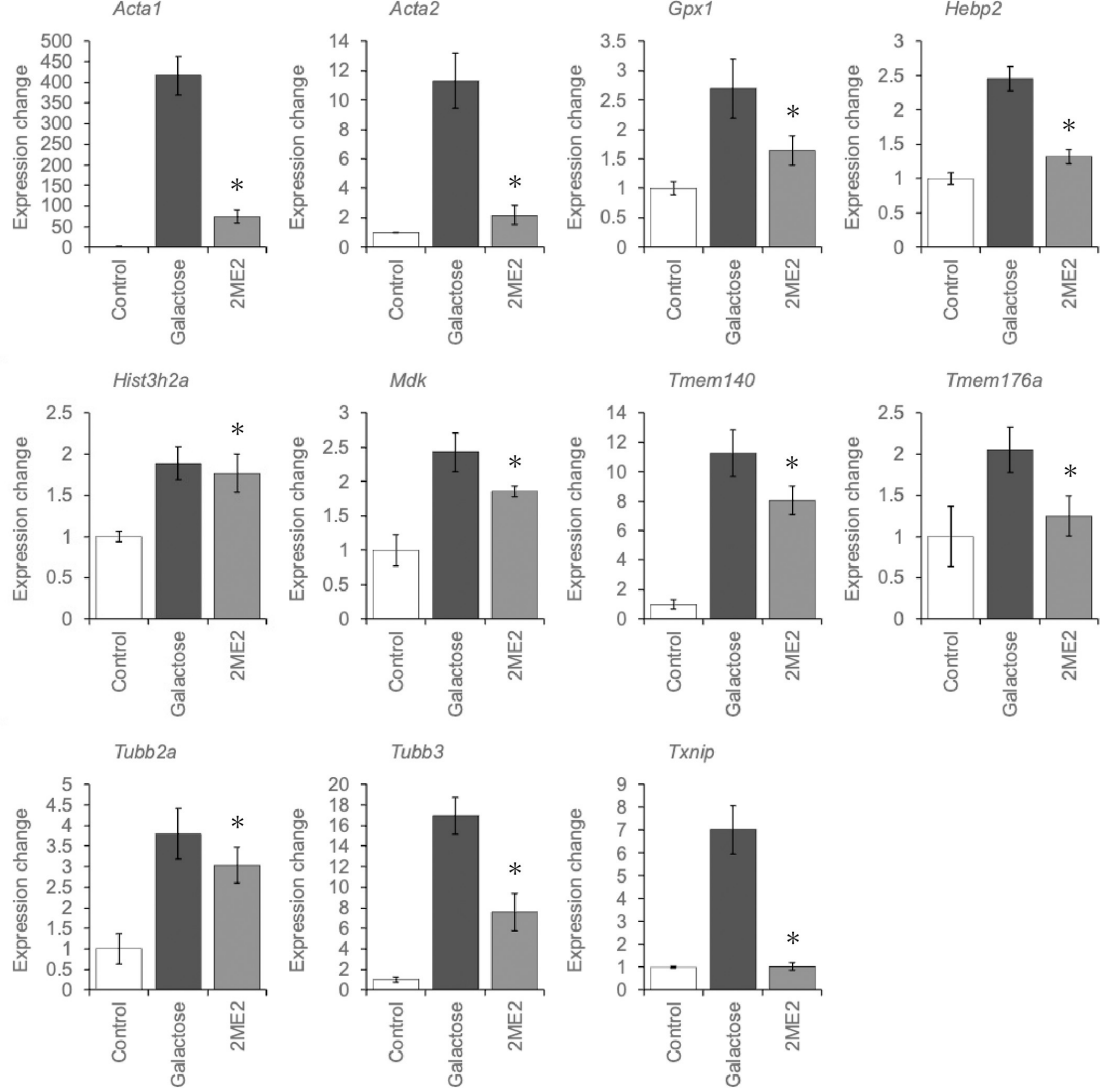
RT-qPCR analysis of genes with altered expression levels. RT-qPCR on 43 genes selected from the microarray analysis, Results are shown as target gene mRNA levels normalized by *Gapdh* mRNA levels. Data are expressed as the mean ± SE. Asterisks indicate more than a 10% decrease in expression between the galactose and 2ME2 groups compared with the amount of increase between the control and galactose groups.

In order to further narrow down the number of genes with a large number of genes for RT-qPCR, we extracted 80 genes, the expression levels of which were decreased by more than two-fold between the Galactose and 2ME2 groups and increased by more than two-fold between the Control and Galactose groups. After excluding functionally unknown genes, ribosomal-protein genes, and genes with challenging primer design, 43 genes were selected for expression level quantification by RT-qPCR (S2 Table). Thirty-two genes were not consistent with the microarray results and eleven genes showed more than a 10% decrease in expression between the galactose and 2ME2 groups compared with the amount of increase between the control and galactose groups, consistent with the microarray results (S3 Fig and Fig 3).

### Functional analysis of genes involved in opacity decrease

**Fig 4:**
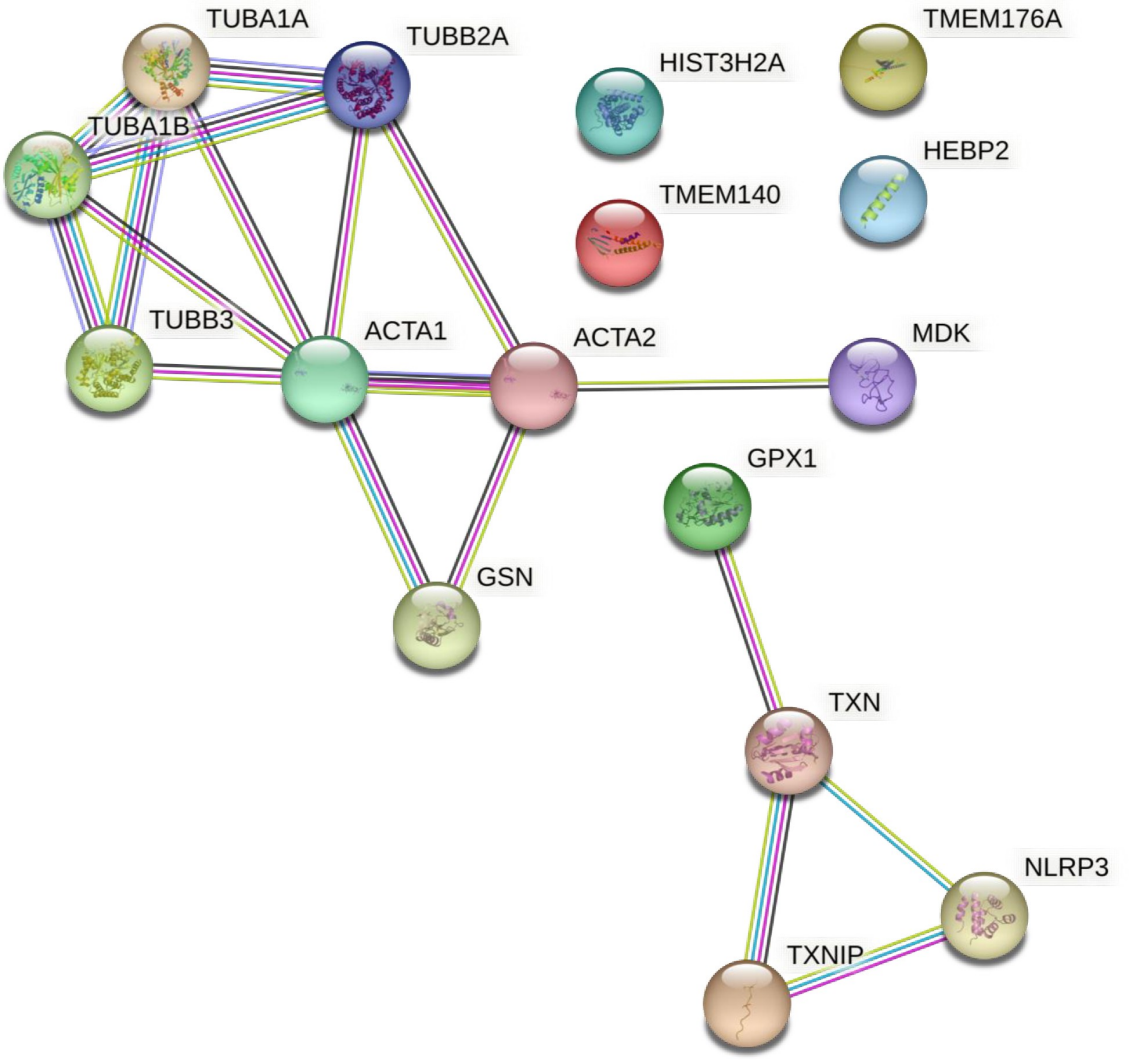
STRING protein interaction analysis. STRING analysis of 11 genes, expression changes were confirmed by RT-qPCR, and their predicted functional partners. The color of each edge shows the type of relationship in the following manner: light blue = “from curated databases,” dark purple = “experimentally determined,” green = “text mining,” black = “co-expression,” and light purple = “protein homology”.

**Fig 5:**
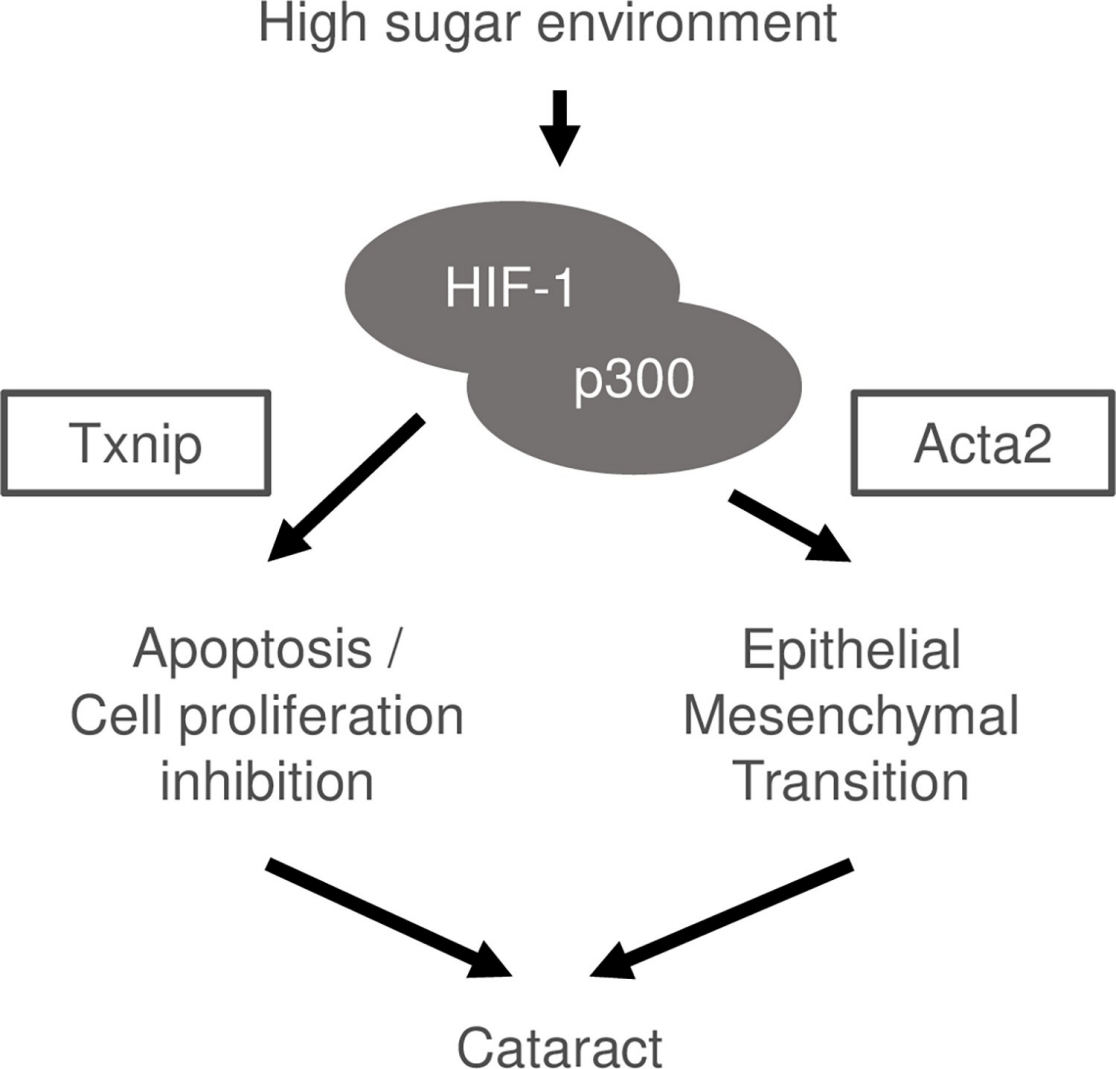
Predicted mechanism of HIF-1-induced diabetic cataract. HIF-1 is induced in high-sugar environments and forms a complex with p300. Subsequently, cataract is caused by EMT induced increase in Acta2 expression, Txnip-induced apoptosis, and inhibition of cell proliferation.

To investigate the functions of these 11 genes, for which expression changes were identified by RT-qPCR, we conducted protein-protein interaction analysis using STRING (S4 Fig). Among them, ACTA1, ACTA2, MDK, TUBB2A, and TUBB3 formed a cluster. ACTA2, situated at the center of the cluster, is a recognized mesenchymal cell marker, [44] and ACTA1, an actin-binding protein, belongs to the actin family of cytoskeletal proteins [45]. MDK is a heparin-binding growth factor and has been reported to promote EMT in cancer cells [46]. TUBB2A and TUBB3, known as β-tubulin, are cytoskeletal components [47]. Thus, suppression of the upregulation of EMT and cytoskeletal-component genes may have improved the opacity phenotype. We further exploited STRING’s ability to add predicted functional partners to investigate putative participating proteins in this network (Fig 4). Interestingly, a cluster involving GPX1 and TXNIP, which were also identified by RT-qPCR, was generated. *Gpx1* encodes a glutathione peroxidase involved in the degradation of hydrogen peroxide, and its expression may have increased upon oxidative stress [48]. TXNIP regulates the intracellular redox state as a thioredoxin-binding protein and is involved in the inhibition of cell proliferation and promotion of apoptosis [49, 50]. Therefore, the HIF-1 inhibitor, 2ME2, may have decreased lens opacity by preventing Txnip-mediated cell growth inhibition, apoptosis, and EMT induced increase in Acta2 expression (Fig 5).

## Discussion

Diabetic cataract is a high-risk disease that occurs even in young people, and its current mainstream treatment involves surgically removing the lens and replacing it with an intraocular lens (IOL). Although drugs such as glycation inhibitors and antioxidants have been shown to prevent cataracts, no drugs have yet achieved fundamental therapy [51, 52]. While AR inhibitors targeting the primary cause of diabetic cataract have shown efficacy in animal experiments, their side effects have not been characterized [53]. Therefore, in this study, we examined whether HIF-1 inhibition could decrease lens opacity in an *ex vivo* model, in which opacity is induced by culturing lenses in galactose-containing medium.

HIF-1 is a hypoxia-induced transcription factor. In recent years, disease therapies targeting HIF-1 have attracted attention. HIF-prolyl hydroxylase (HIF-PH) inhibitors, which activate HIF-1, are used to treat chronic kidney disease by enhancing endogenous erythropoietin production [54]. In addition, experimental inhibition of HIF-1 has been shown to suppress cancer and inflammatory diseases [55]. Conversely, the relationship between cataracts and HIF-1 remains unclear. In age-related cataract, downregulation of Heat shock transcription factor 4 isoform b (HSF4b), which contributes to lens transparency maintenance, is believed to reduce HIF-1α levels, potentially causing cortical and nuclear cataracts [30]. Vitamin C activates PHD, which causes HIF-1α degradation, and when added to human lens epithelial cells (HLEC), it suppresses EMT, which contributes to posterior capsule opacification (PCO) [31].

In this study, rat lenses were cultured in galactose-containing medium to induce opacity. Addition of the HIF-1 inhibitors, 2ME2, YC-1, and Bavachinin, could decrease the opacity formed in the lens cortex (Fig 1B and S1 Fig). We have previously prepared lens tissue sections of galactose samples and samples in which the addition of an inhibitor reduced opacity [56]. Observations showed that the lens cortex collapsed in the galactose sample, whereas in the sample with the inhibitor and reduced opacity, the collapsed cells were pushed into the interior by newly created cells, and the collapse of the lens cortex was apparently almost completely eliminated. Therefore, we believe that the disruption of the lens cortex caused by galactose was normalized by the addition of the HIF-1 inhibitor, and the opacity was decreased.

Next, we conducted comprehensive gene expression analysis to identify genes related to cataract therapy. 2ME2 treatment decreased lens opacity, and genes for which expression increased upon galactose treatment and decreased upon inhibitor treatment were extracted. RT-qPCR was used to confirm changes in their expression levels, and 11 genes were identified (Fig 3). Among them, a cluster of genes related to the cytoskeleton and EMT were induced by opacification and inhibited by 2ME2 (S4 Fig).

EMT is induced by TGF-β signaling, which leads to increased cell motility and accumulation of extracellular matrix, suggesting that it is related to cell invasion and fibrosis [57]. EMT is also involved in the pathogenesis of diabetic cataract [58]. ACTA2 encodes α-smooth muscle actin (α-SMA), a mesenchymal cell marker [59]. Under high-glucose conditions, LEC upregulates α-SMA, leading to EMT [60]. Furthermore, HIF-1α has been shown to promote EMT in various types of tumors and fibrotic diseases [61]. After induction of EMT by TGF-β, HIF-1α translational inhibition decreased α-SMA levels in HLEC [62], suggesting that increased *Acta2* expression under galactose conditions, which known to induced by EMT, could contribute to cortical cataract development. Therefore, reduced *Acta2* expression in the presence of HIF-1 inhibitors is expected to lead to decreased opacity.

Next, we used STRING to analyze protein-protein interactions, including the predicted related factors, and generated a cluster that contained TXNIP (Fig 4). TXNIP binds to and inhibits thioredoxin, which possesses antioxidant activity [49], responding to endoplasmic reticulum stress, and promotes apoptosis [50]. In fact, increased apoptosis of lens LEC has been implicated in the development of diabetic cataract in both human and animal models [63, 64]. TXNIP is induced by oxidative and high-glucose stresses [65, 66], and its expression was higher in the Galactose than in the Control and 2ME2 groups. This suggests that HIF-1 inhibitors may decrease opacity by suppressing apoptosis and activating cell proliferation. In addition, as TXNIP is thought to be involved in EMT [67], inhibition of both EMT and apoptosis may be important for reversing lens opacification (Fig 5).

There are multiple HIF-1 inhibitors, each with different targets. We find the presence or absence of a decrease in lens opacity was observed depending on the different targets within HIF-1 inhibition. The three HIF-1 inhibitors that showed a decrease in lens opacity in this study are 2ME2, YC-1, and Bavachinin. 2ME2 depolymerizes microtubules in tumor cells by interacting with colchicine binding site on β-tubulin, leading to the protein-level inhibition of HIF-1α downstream [34]. However, the association between microtubule depolymerization and the inhibitory mechanism of HIF-1α at the protein level is not yet clear at this stage. In the gene expression analysis, the expression of cytoskeleton-related genes was decreased in the samples treated with 2ME2 compared to the galactose-treated samples. This observation suggests that the suppression of cytoskeletal remodeling and the regulation of EMT could contribute to the reduction of lens opacity. However, in this experiment, the addition of ELR510444, which suppresses HIF-1α protein levels by depolymerizing microtubules as in 2ME2, did not reduce lens opacity [39]. Based on the results of gene expression analysis, we believe that 2ME2 reduced lens opacity by decreasing gene expression of oxidative stress-related genes as well as cytoskeleton-related genes. YC-1 enhances the binding of Factor Inhibiting HIF (FIH) to the C-terminal transactivation domain (CAD) of HIF-1α, dissociates the binding of p300, and contributes to the suppression of HIF-1 function [35]. Similarly, YC-1 inhibits the accumulation of HIF-1α protein, suggesting that YC-1 suppresses HIF-1 activity by multiple targets [68]. Bavachinin is thought to promote HIF-1α degradation by increasing its interaction with VHL [36]. Bavachinin is also known as a PPAR agonist, and some PPAR agonists in our laboratory have been shown to reduce opacity in galactose-induced cataracts [Unpublished data]. Therefore, targeting multiple targets in HIF-1 inhibition may have decreased lens opacity.

We have previously reported that histone acetyltransferase (HAT) and ATM inhibitors decrease opacity in the galactose-induced cataract model [17, 56]. Among HAT inhibitors, TH1834, which targets TIP60, has exhibited therapeutic effects by decreasing opacity [56]. Furthermore, ATM is activated by autophosphorylation through acetylation by TIP60 [69]. In fact, addition of AZD0156 and KU55933, which inhibit ATM, resulted in decreased opacity similar to TH1834 [17]. Additionally, HIF-1α is stabilized by phosphorylation by ATM [18]. In this study, the addition of HIF-1 inhibitors decreased opacity. This suggests that the TIP60-ATM-HIF-1 pathway may be involved in both the formation and reversal of galactose-induced cataract.

We also compared genes exhibiting altered expression levels specifically in RT-qPCR experiments upon HAT and ATM inhibition (opacity decrease) with 11 genes displaying altered expression levels in this study. Common genes with regards to HAT inhibitors were *Acta1*, *Acta2*, *Hebp2*, and *Mdk*. Regarding ATM inhibitors, the common genes identified were *Acta1*, *Acta2*, and *Tubb3*. Furthermore, common genes among the analyses of 2ME2, HAT, and ATM inhibitors were *Acta1* and *Acta2*. This suggested that *Acta1* is involved in the regulation of the cytoskeleton, *Acta2* is related to EMT, and LEC disruption is a common pathogenic mechanism in galactose-induced cataractogenesis. In addition, regulation of EMT and the cytoskeleton through the above inhibitors could induce cell differentiation of normal LEC and decrease opacity. Conversely, our analysis of HAT and ATM inhibitors showed no common genes involved in apoptosis, suggesting that further investigation is needed to determine whether apoptosis is suppressed by these inhibitors.

In this study, we found that HIF-1 inhibition decreased opacity in a galactose-induced cataract model. Furthermore, gene expression analysis suggested that HIF-1 inhibition decreased opacity by suppressing EMT and apoptosis. In future, drug treatment targeting HIF-1 and downstream factors will be important for cataract therapy.

## Supporting information

S1 Fig Effect of HIF-1 inhibitors on galactose-induced cataract. Microscopic photographs of lenses incubated with HIF-1 inhibitors. The upper panel shows the results of culturing rat lenses in 30 mM galactose-containing medium for 2–3 days. The lower panel shows the result of adding HIF-1 inhibitor to galactose-containing medium after photographing the lens in the upper panel and culturing for 2–3 days. Days in the upper left panel indicates total incubation time.
(PDF)

S2 Fig Lens photographs used for opacity quantification. Upper panel shows lens photographs before and lower panel shows lens photographs after addition of inhibitor. In addition to the lens photographs in Fig 1B, a total of three samples were used for opacity quantification. Days in the upper left panel indicates total incubation time, "q" indicates samples RT-qPCR, and "M" indicates samples used for microarray analysis.
(PDF)

S3 Fig Results of RT-qPCR analysis. RT-qPCR on 32 genes which didn’t alter. Results are shown as target gene mRNA levels normalized by *Gapdh* mRNA levels. Data are expressed as the mean ± SE.
(PDF)

S4 Fig Results of protein-protein interaction analysis. Results of STRING analysis of 11 genes whose expression variation was confirmed by RT-qPCR (https://version-11-5.string-db.org/). Organisms selected were *Homo sapiens*. The color of each edge shows the type of relationship in the following manner: light blue = "from curated databases"; dark purple = "experimentally determined"; green = "text mining"; black = "co-expression"; and light purple = "protein homology”.
(PDF)

S1 Table List of primer used for real-time RT-qPCR. (DOCX)

S2 Table List of 43 genes for which RT-qPCR was performed. (DOCX)

S1 Dataset List of 186 genes that were increased more than 2-fold from control to galactose and decreased more than 1.5-fold from galactose to 2ME2. The ten columns on the right of Gene name show the signal value (log2) in each sample. Average indicates the mean value of replicate samples. The column for Control vs Galactose indicates the number of fold increase in expression from Control to Galactose. The column of Galactose vs. 2ME2 shows the number of fold decrease in expression from Galactose to 2ME2.
(XLSX)
