# Supplementary figures and images for: HIF-1 inhibition reverses opacity in a rat model of galactose-induced cataract
Source: PLoS One. 2024 Feb 28;19(2):e0299145. doi: 10.1371/journal.pone.0299145 (PMC10901314; doi:10.1371/journal.pone.0299145)

# YC-1

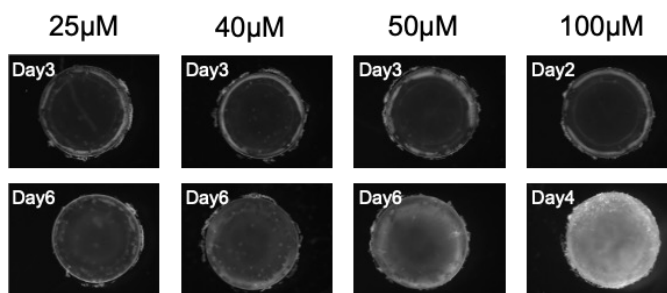

# Bavachinin

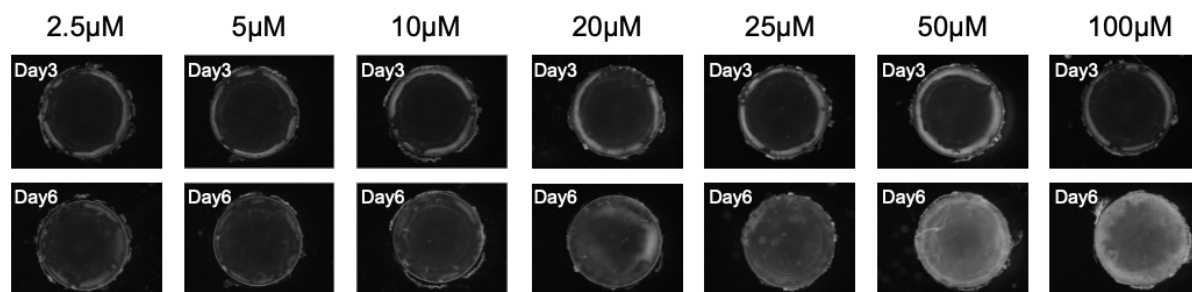

# BAY-87-2243

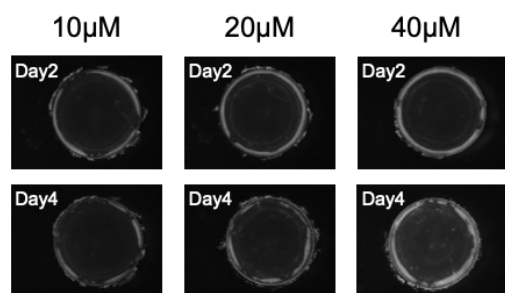

# Chetomin

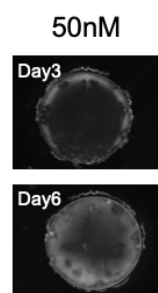

# ELR510444

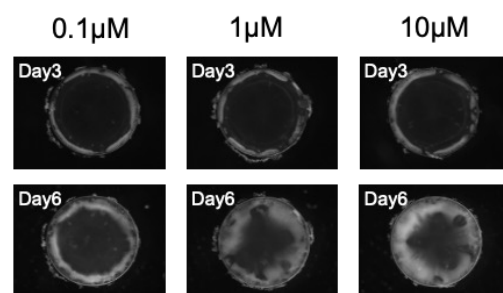

# KC7F2

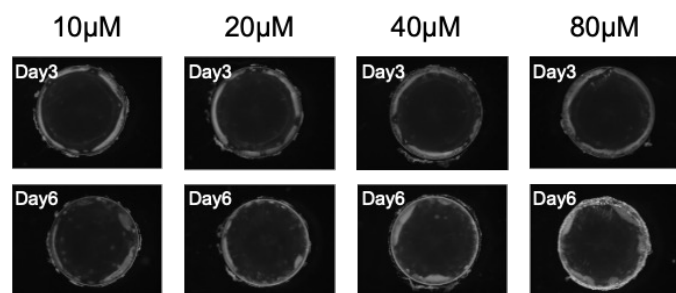

# PX-478

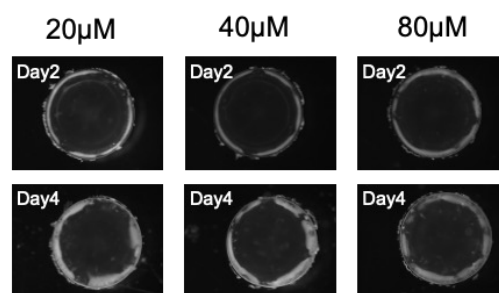

# Topotecan

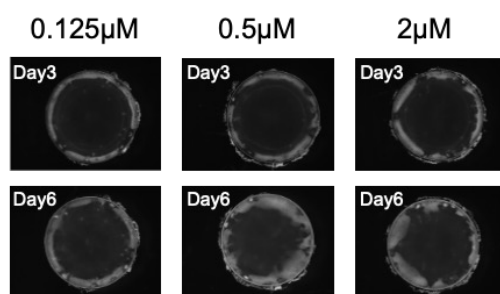

# Vitexin

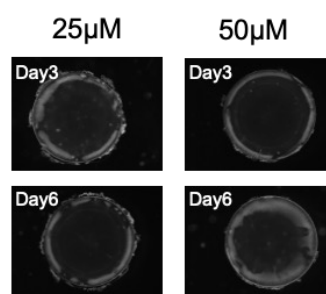

Supplement: S1 Fig — Microscopic photographs of lenses incubated with HIF-1 inhibitors. The upper panel shows the results of culturing rat lenses in 30 mM galactose-containing medium for 2–3 days. The lower panel shows the result of adding HIF-1 inhibitor to galactose-containing medium after photographing the lens in the upper panel and culturing for 2–3 days. Days in the upper left panel indicates total incubation time. (PDF) [file pone.0299145.s001.pdf]

Galactose

2-Methoxyestradiol (2ME2)  
10 $\mu$ M

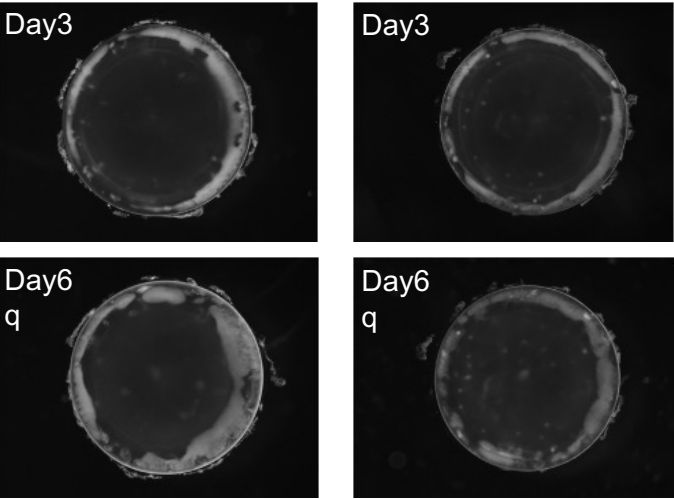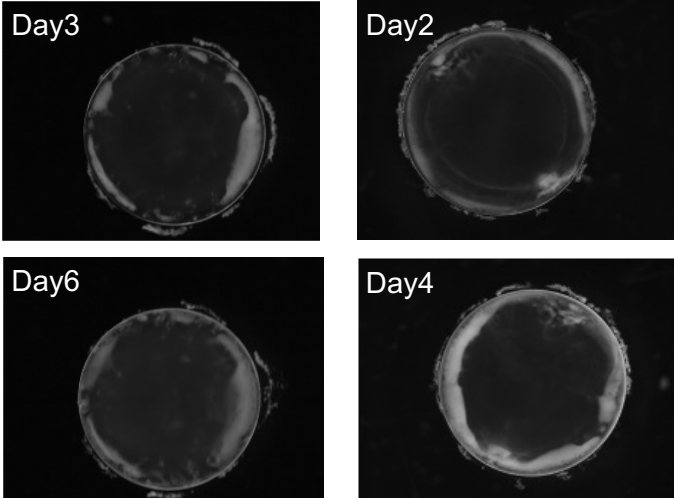

2-Methoxyestradiol (2ME2)  
20 $\mu$ M

2-Methoxyestradiol (2ME2)  
40 $\mu$ M

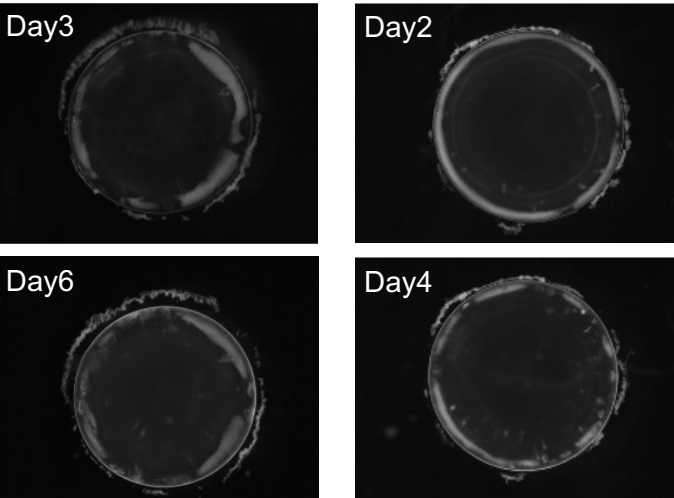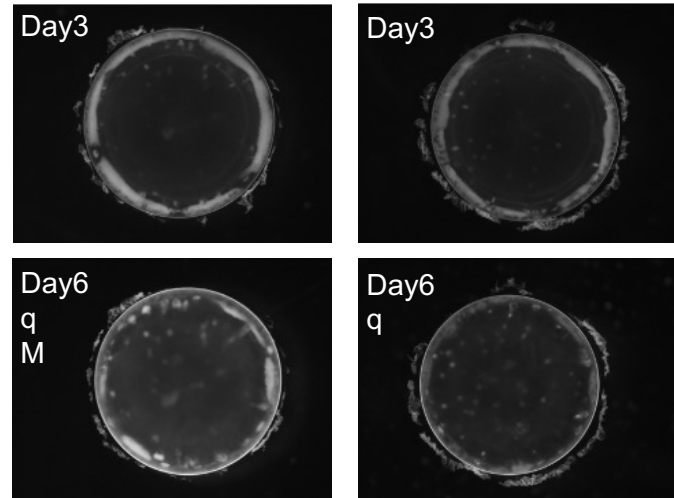

Supplement: S2 Fig — Upper panel shows lens photographs before and lower panel shows lens photographs after addition of inhibitor. In addition to the lens photographs in Fig 1B, a total of three samples were used for opacity quantification. Days in the upper left panel indicates total incubation time, "q" indicates samples RT-qPCR, and "M" indicates samples used for microarray analysis. (PDF) [file pone.0299145.s002.pdf]

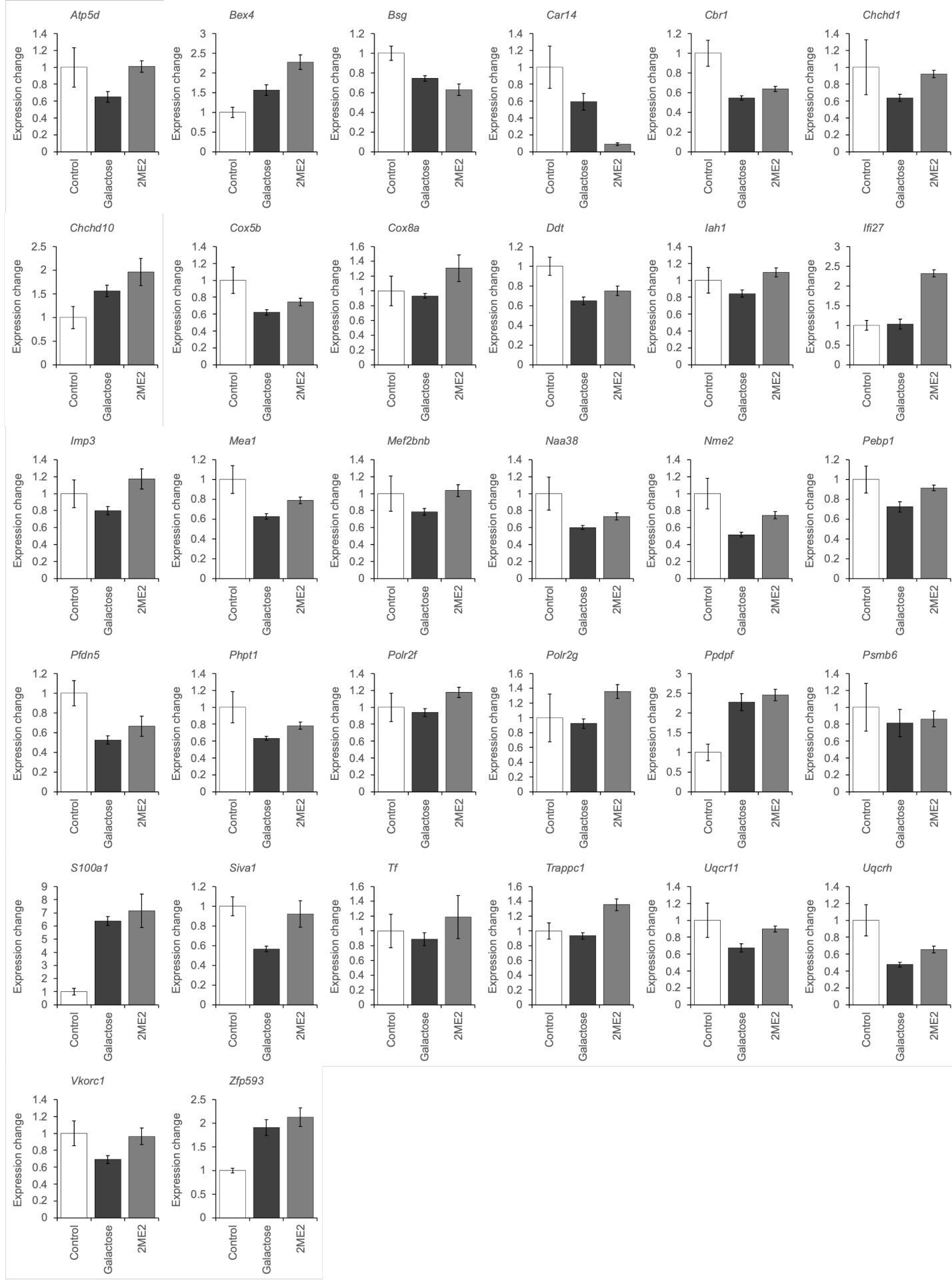

Supplement: S3 Fig — RT-qPCR on 32 genes which didn’t alter. Results are shown as target gene mRNA levels normalized by Gapdh mRNA levels. Data are expressed as the mean ± SE. (PDF) [file pone.0299145.s003.pdf]

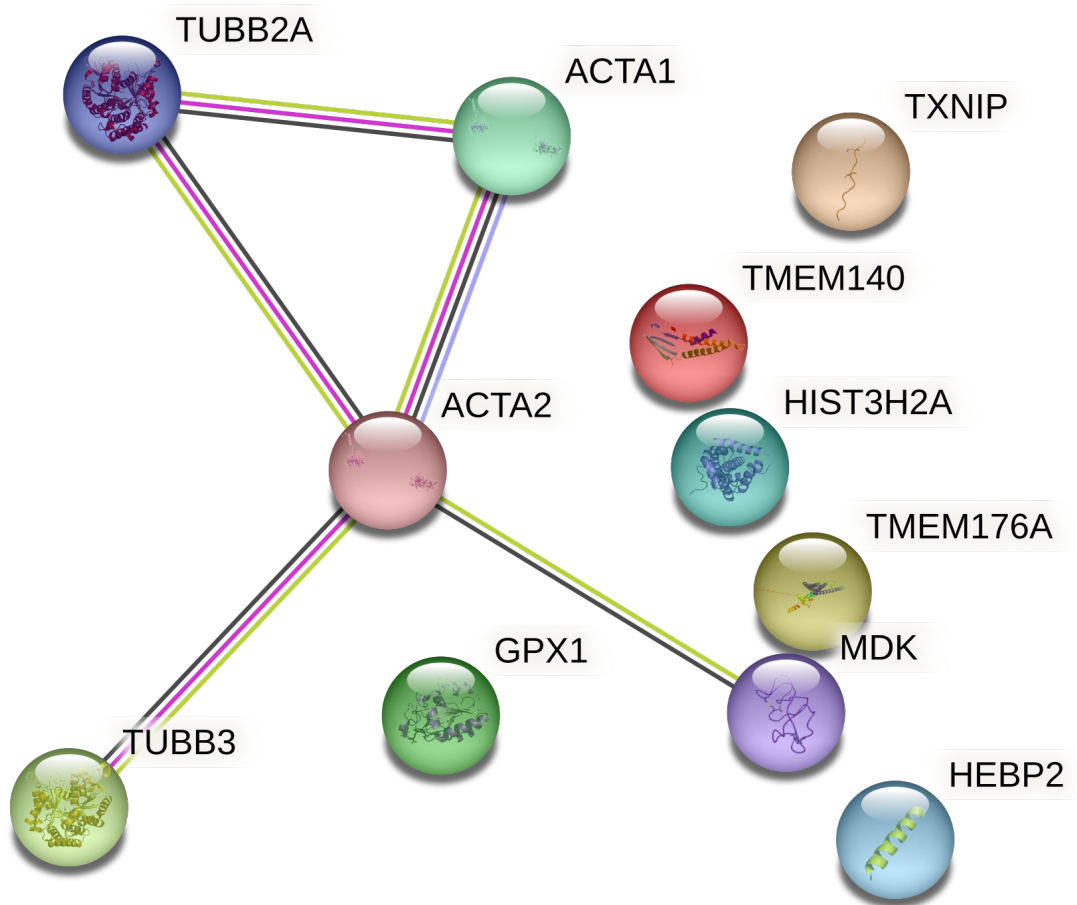

Supplement: S4 Fig — Results of STRING analysis of 11 genes whose expression variation was confirmed by RT-qPCR (https://version-11-5.string-db.org/). Organisms selected were Homo sapiens. The color of each edge shows the type of relationship in the following manner: light blue = "from curated databases"; dark purple = "experimentally determined"; green = "text mining"; black = "co-expression"; and light purple = "protein homology”. (PDF) [file pone.0299145.s004.pdf]
