# Supplementary material for: HIF-1 inhibition reverses opacity in a rat model of galactose-induced cataract
Source: PLoS One. 2024 Feb 28;19(2):e0299145. doi: 10.1371/journal.pone.0299145 (PMC10901314; doi:10.1371/journal.pone.0299145)
Supplement: S1 Table — (DOCX) [file pone.0299145.s005.docx]

| ***Primer List*** | | |
| --- | --- | --- |
| **Gene** | **Forward primer** | **Reverse primer** |
| *Gapdh* | 5'-GAGACAGCCGCATCTTCTTGT-3' | 5'-CGACCTTCACCATCTTGTCTATGA-3' |
| *Acta1* | 5'-TCAGGCGGTGCTGTCTCTCT-3' | 5'-TCCCCAGAATCCAACACGAT-3' |
| *Acta2* | 5'-ACCAGGGAGTGATGGTTGGA-3' | 5'-CGCTTCGTCCCCCACATA-3' |
| *Atp5d* | 5'-TGGGCTGGTAGTGGTTCATG-3' | 5'-GAGCCGCTGCTCACAAAATAC-3' |
| *Bex4* | 5'-ATGAGGCCTTACACGCGTTT-3' | 5'-ATGAGGCAAAAGTCGTAATGATTG-3' |
| *Bsg* | 5'-TTGGCCACCGCTGGAT-3' | 5'-ATCGGGCAGCGTGTCTTC-3' |
| *Car14* | 5'-CTCAGAAGCCTCAGGGACTG-3' | 5'-TTGTGAGCGAGCCATTGTAG-3' |
| *Cbr1* | 5'-AGGACAAGATCCTCCTGAATGC-3' | 5'-CCTGCCATGTCGGTTCTGA-3' |
| *Chchd1* | 5'-CGAGGCGTGCAGGAAAGA-3' | 5'-TCCTGAGCCTTGGAAGAACAA-3' |
| *Chchd10* | 5'-ACCCTGTGTGAGGGCTTCAG-3' | 5'-GAGCTCAGACCGTGATTGTATTTG-3' |
| *Cox5b* | 5'-GCCCCAACTGTGGAACACAT-3' | 5'-GTGGGCTCAGTGGACCATTT-3' |
| *Cox8a* | 5'-CACTTCCTGCTTCGTGTGTTG-3' | 5'-CTTGTAGCTTTCCAGGTGTGACA-3' |
| *Ddt* | 5'-ATCCTGGACAAACCCGAAGA-3' | 5'-AGGTCATGCCCGGTCGTAT-3' |
| *Gpx1* | 5'-GCGCTGGTCTCGTCCATT-3' | 5'-TGGTGAAACCGCCTTTCTTT-3' |
| *Hebp2* | 5'-CCGCCTTGTGGCTCAAAC-3' | 5'-ACGCACGGCCCTGAAA-3' |
| *Hist3h2a* | 5'-CGGCCCGCGATAATAAGAA-3' | 5'-TTGCGGATGGCCAACTG-3' |
| *Iah1* | 5'-CCTCGCGTGTTGCTCTTTG-3' | 5'-CCATCCACCCTGCTGGAA-3' |
| *Ifi27* | 5'-TGCACTTGGATCTGCATTGG-3' | 5'-GGCAGGGAGGCTGGATAAAA-3' |
| *Imp3* | 5'-AGCGGCACGTGTTGGAGTA-3' | 5'-CTCGCTAGGCATCAAGATCAAA-3' |
| *Mdk* | 5'-GGTGCCCTGCAACTGGAA-3' | 5'-CCCCAGCTCTCAAACTTGTATTTG-3' |
| *Mea1* | 5'-TGAGCAGCCACAGCTCCAT-3' | 5'-CCAGCCATCGTCCTCTTCAC-3' |
| *Mef2bnb* | 5'-GCCAGGGCGCCATATACAC-3' | 5'-GCTGTCCACCAAGCTCTTCAC-3' |
| *Naa38* | 5'-GCGCCGACCCCAAAA-3' | 5'-GGCCCTGGGACCTCCTTT-3' |
| *Nme2* | 5'-GAAGCCAGGCACCATTCGT-3' | 5'-ACTGCCGTGAATGATGTTCCT-3' |
| *Pebp1* | 5'-TGGCACTGTCCTCTCCGAAT-3' | 5'-GGTGCAGACCTGTGTCTTTGG-3' |
| *Pfdn5* | 5'-CAGGAGAAGCATGCCATGAA-3' | 5'-TGCTGAATCTTCTGGCTCATCA-3' |
| *Phpt1* | 5'-TCAGAGCCAGGACAGGAAGATAC-3' | 5'-CTGGGCACGGCCGTAA-3' |
| *Polr2f* | 5'-TGAGGAGGAGGGTCAGGAAA-3' | 5'-GGCCTGCGGTCGTTCAC-3' |
| *Polr2g* | 5'-TGGTGGATGCTGTGGTCACT-3' | 5'-TGGGCCCAATTTCTGTGAAA-3' |
| *Ppdpf* | 5'-GGAAATCCACCCTCCCATTC-3' | 5'-TGCCGAGTGCTCAGGAGACT-3' |
| *Psmb6* | 5'-GCGGCAGGTGCTTTTGG-3' | 5'-GGTGGCAAAGTGGAGATGGT-3' |
| *S100a1* | 5'-CATCCCACCTGTATCTCCCTATG-3' | 5'-CGCCTTTGGTGCATGTTG-3' |
| *Siva1* | 5'-TGTTCGCCGAGCGCTACT-3' | 5'-TGGAAAAGGAGCTGCTTGGT-3' |
| *Tf* | 5'-GAAGGCAGCCCGGGTTAG-3' | 5'-GTCACCTTTCCAAAATAAATCCTTCT-3' |
| *Tmem140* | 5'-AAGAACCACTGGGAGAACTGCTA-3' | 5'-TGAACCCTTGCCATCCCATA-3' |
| *Tmem176a* | 5'-CGGGATCGTGGCTATGCT-3' | 5'-CCACCCCGTTTCTTTTGAAG-3' |
| *Trappc1* | 5'-ACCGGAATGGAGTGTGTCTACA-3' | 5'-GGGATCCCTGCTTGCTTCTT-3' |
| *Tubb2a* | 5'-GCCAATGCGGCAACCA-3' | 5'-GCCATGCTCATCGCTTATCA-3' |
| *Tubb3* | 5'-GGGCCTTTGGACACCTATTCA-3' | 5'-CCCTTTGGCCCAGTTGTTG-3' |
| *Txnip* | 5'-TGACCGTGCAGCCTGTGA-3' | 5'-CGCTCCCACGGTTAGTCAAC-3' |
| *Uqcr11* | 5'-CAGCGATGCTCAGCAGATTTC-3' | 5'-ATCCAGTTTTTGGCCAGTTCTC-3' |
| *Uqcrh* | 5'-TGCATGCACGGGATCACT-3' | 5'-CTGCATGTTTACTTCAAGCTTTTGA-3' |
| *Vkorc1* | 5'-TTGCGGCTTGCACTATGC-3' | 5'-CGTGCAGTGCGTACAGTGAGA-3' |
| *Zfp593* | 5'-CCGATCCAAAGACCACAAGAA-3' | 5'-CTCTTCCTGACTGTAGGGTTCGA-3' |
