# Supplementary material for: HIF-1 inhibition reverses opacity in a rat model of galactose-induced cataract
Source: PLoS One. 2024 Feb 28;19(2):e0299145. doi: 10.1371/journal.pone.0299145 (PMC10901314; doi:10.1371/journal.pone.0299145)
Supplement: S2 Table — (DOCX) [file pone.0299145.s006.docx]

| **Gene** | **Description** |
| --- | --- |
| *Acta1* | actin, alpha 1, skeletal muscle (Acta1). |
| *Acta2* | actin, alpha 2, smooth muscle (Acta2). |
| *Atp5d* | ATP synthase, H+ transporting, mitochondrial F1 complex, delta subunit (Atp5d). |
| *Bex4* | brain expressed, X-linked 4 (Bex4). |
| *Bsg* | basigin (Bsg). |
| *Car14* | carbonic anhydrase 14 (Car14). |
| *Cbr1* | carbonyl reductase 1 (Cbr1). |
| *Chchd1* | coiled-coil-helix-coiled-coil-helix domain containing 1 (Chchd1). |
| *Chchd10* | coiled-coil-helix-coiled-coil-helix domain containing 10 (Chchd10). |
| *Cox5b* | cytochrome c oxidase subunit Vb (Cox5b). |
| *Cox8a* | cytochrome c oxidase subunit VIIIa (Cox8a). |
| *Ddt* | D-dopachrome tautomerase (Ddt). |
| *Gpx1* | glutathione peroxidase 1 (Gpx1). |
| *Hebp2* | heme binding protein 2 (Hebp2). |
| *Hist3h2a* | histone cluster 3, H2a (Hist3h2a). |
| *Iah1* | isoamyl acetate-hydrolyzing esterase 1 homolog (Iah1). |
| *Ifi27* | interferon, alpha-inducible protein 27 (Ifi27). |
| *Imp3* | IMP3, U3 small nucleolar ribonucleoprotein (Imp3). |
| *Mdk* | midkine (Mdk). |
| *Mea1* | male-enhanced antigen 1 (Mea1). |
| *Mef2bnb* | MEF2B neighbor (Mef2bnb). |
| *Naa38* | N(alpha)-acetyltransferase 38, NatC auxiliary subunit (Naa38). |
| *Nme2* | NME/NM23 nucleoside diphosphate kinase 2 (Nme2). |
| *Pebp1* | phosphatidylethanolamine binding protein 1 (Pebp1). |
| *Pfdn5* | prefoldin subunit 5 (Pfdn5). |
| *Phpt1* | phosphohistidine phosphatase 1 (Phpt1). |
| *Polr2f* | polymerase (RNA) II (DNA directed) polypeptide F (Polr2f). |
| *Polr2g* | polymerase (RNA) II (DNA directed) polypeptide G (Polr2g). |
| *Ppdpf* | pancreatic progenitor cell differentiation and proliferation factor (Ppdpf). |
| *Psmb6* | proteasome (prosome, macropain) subunit, beta type 6 (Psmb6). |
| *S100a1* | S100 calcium binding protein A1 (S100a1). |
| *Siva1* | SIVA1, apoptosis-inducing factor (Siva1). |
| *Tf* | transferrin (Tf). |
| *Tmem140* | transmembrane protein 140 (Tmem140). |
| *Tmem176a* | transmembrane protein 176A (Tmem176a). |
| *Trappc1* | trafficking protein particle complex 1 (Trappc1). |
| *Tubb2a* | tubulin, beta 2A class IIa (Tubb2a). |
| *Tubb3* | tubulin, beta 3 class III (Tubb3). |
| *Txnip* | thioredoxin interacting protein (Txnip). |
| *Uqcr11* | ubiquinol-cytochrome c reductase, complex III subunit XI (Uqcr11). |
| *Uqcrh* | ubiquinol-cytochrome c reductase hinge protein (Uqcrh). |
| *Vkorc1* | vitamin K epoxide reductase complex, subunit 1 (Vkorc1). |
| *Zfp593* | zinc finger protein 593 (Zfp593). |
